# Mechanical Properties and Damage Constitutive Model of Saturated Sandstone Under Freeze–Thaw Action

**DOI:** 10.3390/ma17235905

**Published:** 2024-12-02

**Authors:** Meimei Feng, Xiaoxiao Cao, Taifeng Wu, Kangsheng Yuan

**Affiliations:** 1 State Key Laboratory of Intelligent Construction and Healthy Operation and Maintenance of Deep Underground Engineering, China University of Mining and Technology, Xuzhou 221116, China; fengmeimei@cumt.edu.cn; 2 School of Mechanics & Civil Engineering, China University of Mining & Technology, Xuzhou 221116, China; ts24030028a31@cumt.edu.cn (T.W.); ts20030220p31@cumt.edu.cn (K.Y.); 3 Laboratory of Rock Engineering & Mining Machinery, Department of Earth Resources Engineering, Kyushu University, Fukuoka 8190395, Japan

**Keywords:** freeze–thaw, water-force coupling, mechanical properties, nuclear magnetic resonance, damage model

## Abstract

In order to investigate the impact of freeze–thaw damage on sandstone under the coupling of ground stress and pore water pressure, three types of porous sandstone were subjected to freezing at different negative temperatures (−5 °C, −10 °C, −15 °C, and −20 °C). Subsequently, hydraulic coupling triaxial compression tests were conducted on the frozen and thawed sandstone. We analyzed the effects of porosity and freezing temperature on the mechanical properties of sandstone under hydraulic coupling and performed nuclear magnetic resonance tests on sandstone samples before and after freezing and thawing. The evolution of the pore structure in sandstone at various freezing and thawing stages was studied, and a statistical damage constitutive model was established to validate the test results. The results indicate that the stress–strain curves of sandstone samples under triaxial compression after a freeze–thaw cycle exhibit minimal changes compared to those without freezing at normal temperature. The peak deviator stress shows a decreasing trend with decreasing freezing temperature, particularly between −5 °C and −10 °C, and then gradually stabilizes. The elastic modulus of sandstone with different porosity decreases with the decrease in freezing temperature, and the decrease is more obvious in the range of −5 °C~−10 °C, decreasing by 2.33%, 6.11%, and 10.5%, respectively. Below −10 °C, the elastic modulus becomes similar to that at −10 °C, and the change tends to stabilize. The nuclear magnetic porosity of sandstone samples significantly increases after freezing and thawing. The smaller the initial porosity, the greater the rate of change in nuclear magnetic porosity after a freeze–thaw cycle. The effects of freeze–thaw damage on the T2 distribution of sandstone with different porosity levels vary. We established a statistical damage constitutive model considering the combined effects of freeze–thaw damage, ground stress, and pore water pressure. The compaction coefficient *K* was introduced into the constitutive model for optimization. The change trend of the theoretical curve closely aligns with that of the test curve, better characterizing the stress–strain relationship of sandstone under complex pressure environments. The research findings can provide a scientific basis for wellbore wall design and subsequent maintenance in complex environments.

## 1. Introduction

Geotechnical engineering construction inevitably faces challenges in complex geological environments, including high groundwater levels and low surrounding rock strength. Sudden events such as water inrushes, mud outbursts, and significant deformations of surrounding rock pose threats to construction safety [1,2]. The freezing method, commonly employed in China, is an effective means to control groundwater [3]. Concurrently, the mechanical properties of rocks are significantly enhanced after freezing compared to their pre-frozen state [4,5]. The deformation and strength characteristics of rocks after thawing, influenced by the combined effects of water pressure and ground stress [6,7], directly impact the design and subsequent maintenance of wellbore walls. Therefore, it is necessary to conduct hydraulic coupled triaxial compression tests on rocks after freeze–thaw cycles, and explore and analyze the mechanical characteristics and influencing factors of sandstone under the coupled effects of water pressure and confining pressure.

Several researchers, both nationally and internationally, have investigated the influence of freeze–thaw cycles on the physical and mechanical properties of rocks. For instance, Khanlari G et al. [8] conducted P-wave velocity, porosity, and uniaxial compressive strength tests on five different types of sandstone after freeze–thaw cycles, studying the impact on the physical and mechanical properties of red sandstone in central Iran. Cao et al. [9] discussed the effects of hydrochemical corrosion and freeze–thaw cycles on the dynamic mechanical properties and microstructure of rocks. Similarly, Gao et al. [10] investigated changes in density, ultrasonic velocity, and both static and dynamic mechanical behavior of sandstone subjected to 10 freeze–thaw cycles at various low temperatures, revealing the effects on sandstone density, P-wave velocity, stress–strain relationships, static and dynamic uniaxial compressive strength, static elastic modulus, and dynamic energy absorption. In related work, Jamshidi et al. [11] examined multiple mechanical indicators of granite post-freeze–thaw cycles, finding the greatest impact on tensile strength and the smallest effect on uniaxial compressive strength. Additional studies focused on the effects of freeze–thaw cycles on soil and rock under varying conditions. Shi et al. [12] quantitatively analyzed the damage degree of red sandstone subjected to freeze–thaw cycles using triaxial compression tests, incorporating mechanical parameters like peak stress, elastic modulus, and Poisson’s ratio, and established a damage constitutive model for freeze–thaw loads. In cold regions, freeze–thaw cycles and water environments usually have a long-term impact on the mechanical effects of rocks. Therefore, many scholars have conducted targeted research on saturated rocks under freeze–thaw cycles. A large number of scholars’ research on freeze–thaw saturated rocks mainly focuses on the changes in physical properties such as rock porosity, density, and wave velocity [13,14,15]. Studying the impact of freeze–thaw cycles on the physical properties of saturated rocks is of great significance for practical engineering. During the freeze–thaw process, the pore water inside the rock expands after freezing, and the frost heave force generated by it causes the cracks inside the rock to gradually expand. In view of this feature, Hou et al. [16] improved the physical properties of sandstone by the liquid nitrogen freeze–thaw method, thereby improving the production capacity of tight sandstone gas. In order to evaluate the durability of actual rock mass engineering such as wellbore walls and tunnels, Huang et al. [17] proposed a discrete element method that can incorporate smaller and different sized particles to evaluate the durability impact of freeze–thaw cycle rock mass engineering.

These studies provide valuable insights into how freeze–thaw cycling affects physical and mechanical properties, but further investigation is required to understand the microstructural damage induced by freeze–thaw processes. In an effort to understand the underlying mechanisms of microstructural damage caused by freeze–thaw action, numerous researchers have employed advanced testing techniques. Liu et al. [18,19] used three-dimensional digital rock technology to analyze microscale pore structure changes in rock samples during freeze–thaw processes under varying confining pressures and temperatures. Their triaxial seepage tests on sandstone, along with acoustic emission monitoring, provided insights into the mechanical and hydraulic behavior of mudstone post-freeze–thaw cycling. In related work, Chen et al. [20] employed triaxial compression tests, acoustic emission monitoring, and mercury intrusion porosimetry to investigate the impact of freeze–thaw cycles on the compressive strength and internal microstructure of sandstone. At the macroscopic level, the failure mode of sandstone shifted from splitting failure to shear failure, while microscopic analysis showed an increase in the proportion of large pores. Song et al. [21] expanded this research by studying the damage mechanical properties of red sandstone with varying saturation levels under freeze–thaw cycles. Using scanning electron microscopy and uniaxial compression tests on red sandstone samples with five different saturation levels, they developed macroscopic statistical damage equations for freeze–thaw failure in rocks with different saturation levels. These studies collectively highlight the need for a deeper understanding of freeze–thaw-induced microstructural damage, as it plays a critical role in the mechanical degradation of rocks. While significant progress has been made in freeze–thaw research, much work remains to be done, particularly in developing constitutive models to quantify freeze–thaw damage. Numerous scholars have sought to characterize freeze–thaw damage using various parameters and have developed constitutive models for rocks subjected to freeze–thaw cycles [22]. For instance, Feng et al. [23] investigated changes in chemical composition, microstructure, and damage mechanical properties of sandstone subjected to freeze–thaw cycles in acidic environments, utilizing X-ray diffraction, scanning electron microscopy, and uniaxial compression tests. They quantified the strength damage of sandstone using longitudinal wave velocity and optimized a statistical damage model. Zheng et al. [24] proposed a novel freeze–thaw damage model for rocks, validated through experiments on Sichuan sandstone and granite under triaxial compression, effectively capturing nonlinear mechanical behavior and incorporating void compaction and confining pressure influences, providing insights into deformation and failure mechanisms under freeze–thaw cycles. Jia et al. [25] developed a mesoscopic damage constitutive model for granite under freeze–thaw cycles (FTCs), based on tests of granite from Northeast China. The study found that the first 20 FTCs caused significant damage, with slower degradation afterward. The model, incorporating mesoscopic parameters and correction coefficients, performed well in uniaxial compression tests.

Research on the impact of freeze–thaw cycles on the fundamental physical and mechanical properties of rocks has been relatively extensive. However, there is a scarcity of studies addressing the mechanical properties of rocks and the evolution of damage under the coupled effects of water and mechanics following rock freeze–thaw cycles. For instance, during the construction process involving the freezing method, engineering rock continues to experience the combined influences of geostress and water pressure. In this study, freeze–thaw tests were conducted on three sandstone samples with different porosities at different negative temperatures, and then hydraulic coupled triaxial compression tests and nuclear magnetic resonance tests were conducted on the same type of sandstone. The study focused on analyzing the effects of freeze–thaw on the mechanical properties of sandstone, and studied the microevolution mechanism of the sandstone pore structure during freeze–thaw cycles. In addition, a statistical damage constitutive model that comprehensively considers the effects of freeze–thaw damage, ground stress, and pore water pressure was established. The research results provide a scientific basis for shaft wall design and subsequent maintenance, especially in special geological environments such as alpine areas, deep mines, and frozen soil areas, where the effects of freeze–thaw cycles on rock mechanical behavior are particularly significant. The constitutive model established through this experiment can provide theoretical support for underground engineering, tunnel construction, shaft wall reinforcement, etc., in these areas, and help predict the mechanical degradation characteristics of rocks under freeze–thaw cycles, providing a basis for the optimization of construction and maintenance plans.

## 2. Materials and Methods

### 2.1. Sample Preparation and Handling

The experiment utilized three different porosity sandstone samples, sourced from a coal mine in western China. According to the specifications set by the International Society for Rock Mechanics (ISRM), rocks with consistent texture and no surface cracks were selected [26]. A cylindrical standard rock sample of diameter 50 mm and height 100 mm was employed, with both ends polished to meet the criteria for parallel accuracy (≤0.05 mm) and vertical accuracy (≤0.25°). To reduce the discreteness of the experimental results, the processed samples underwent acoustic testing, and those exhibiting similar and more stable wave velocities were selected for the experiment.

Preceding the experiment, the sandstone sample was heated to 110 °C in a drying oven and dried for 24 h. Upon removal and cooling, the sample was weighed to determine its dry density. Subsequently, the cooled sandstone sample underwent vacuum saturation in a saturation barrel. The water surface in the barrel exceeded 5 cm above the upper surface of the sample, and the saturation pressure was set at 0.1 MPa. Once no bubbles emerged from the rock sample’s surface and the saturation time exceeded 12 h, the sample was considered saturated. It was then removed and weighed to calculate the saturation density. The initial porosity of sandstone is calculated by the difference between saturated density and dry density. Three sandstone samples with varying porosities were selected to measure basic mechanical properties, such as uniaxial compressive strength (UCS). The fundamental physical parameters are detailed in Table 1.

**Figure 1:**
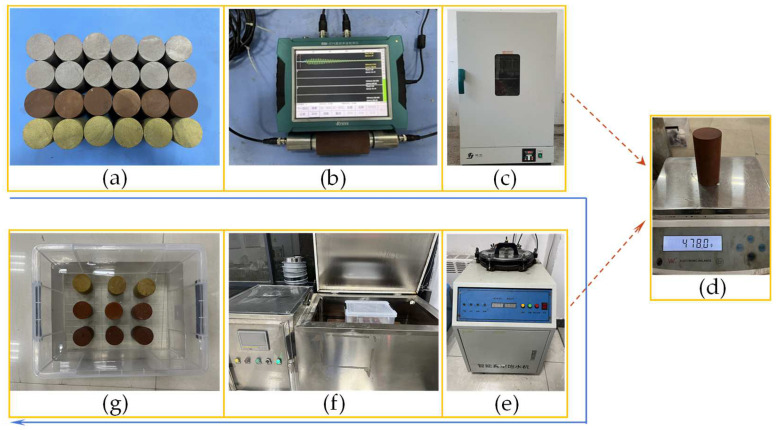
Sample preparation: (**a**) rock samples; (**b**) ultrasonic testing; (**c**) drying oven; (**d**) sample weighing; (**e**) saturation; (**f**) freezing–thawing box; (**g**) thawing of samples.

The freeze–thaw process was conducted on a fully automatic low-temperature freeze–thaw cycle testing machine. Referring to the temperature data of the freezing method construction, we set the freezing temperatures to −5 °C, −10 °C, −15 °C, and −20 °C. To ensure sufficient freezing of the rock samples, the low-temperature constant temperature time was set to 12 h. After the freezing was completed, the frozen sandstone samples were immersed in room-temperature water for melting and backup, and the thawing time was also set to 12 h. The detailed sample processing process is shown in Figure 1. According to the distribution pattern of geostress in China and the feasibility of the experimental system, the pore water pressure [27], horizontal geostress, and vertical geostress of the sample were taken as 3 MPa, 4.5 MPa, and 7.5 MPa, respectively. The cylindrical rock samples of diameter 25 mm and height 50 mm were selected for nuclear magnetic resonance testing under the same conditions as the triaxial compression sample.

### 2.2. Test Equipment and Process

**Figure 2:**
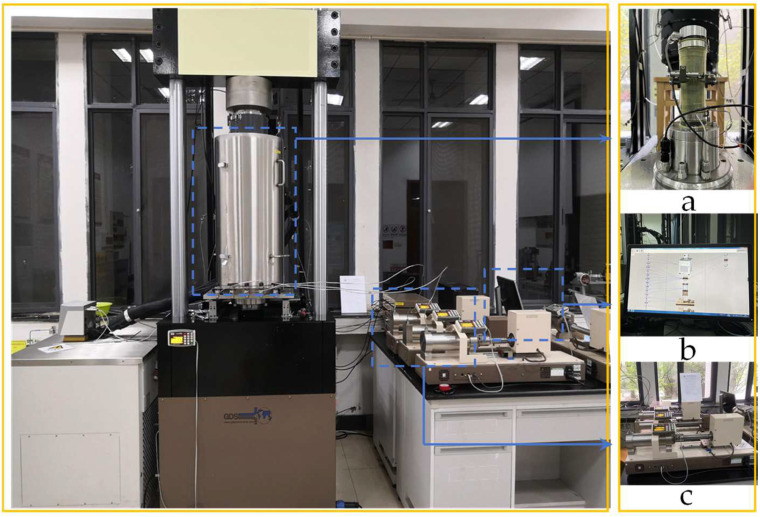
Triaxial test system: (**a**) rock sample installation; (**b**) control and monitoring system; (**c**) pressure control system.

This experiment primarily utilized the high-pressure environment rock and soil triaxial test system (Figure 2), comprising a high-pressure triaxial pressure chamber, a confining water pressure control system, and a control monitoring system. The system is capable of delivering an axial load of up to 400 kN. The confining pressure and water pressure control system can generate a maximum pressure of 64 MPa, employing volume control with an accuracy of 0.1%. Each component can be operated and controlled independently, and they can also be cross-controlled through the control monitoring system. Real-time monitoring of data indicators from each system ensures the stability of various pressures throughout the test process.

The rock sample, having undergone a single freeze–thaw cycle, was placed into the triaxial pressure chamber and connected to the inlet pipeline and displacement sensor. First, the sample was loaded with confining pressure and initial axial pressure, and then observed the stability of the pressure through the control detection system before loading the water pressure. The process of hydraulic loading was quite important and the program was complex. The outlet pressure and inlet pressure were both initially set at 0 MPa, with the target value subsequently adjusted. Once the inlet and outlet volumes were equal, the outlet pressure was set to the target value and maintained for a specific duration to ensure the stability of the internal water pressure within the sample. This allowed the commencement of the test, where axial loading was carried out using the displacement loading method at a loading speed of 0.01 mm/s.

## 3. Experimental Results and Analysis

### 3.1. Deformation Characteristics of Sandstone and Changes in Triaxial Compressive Strength

**Figure 3:**
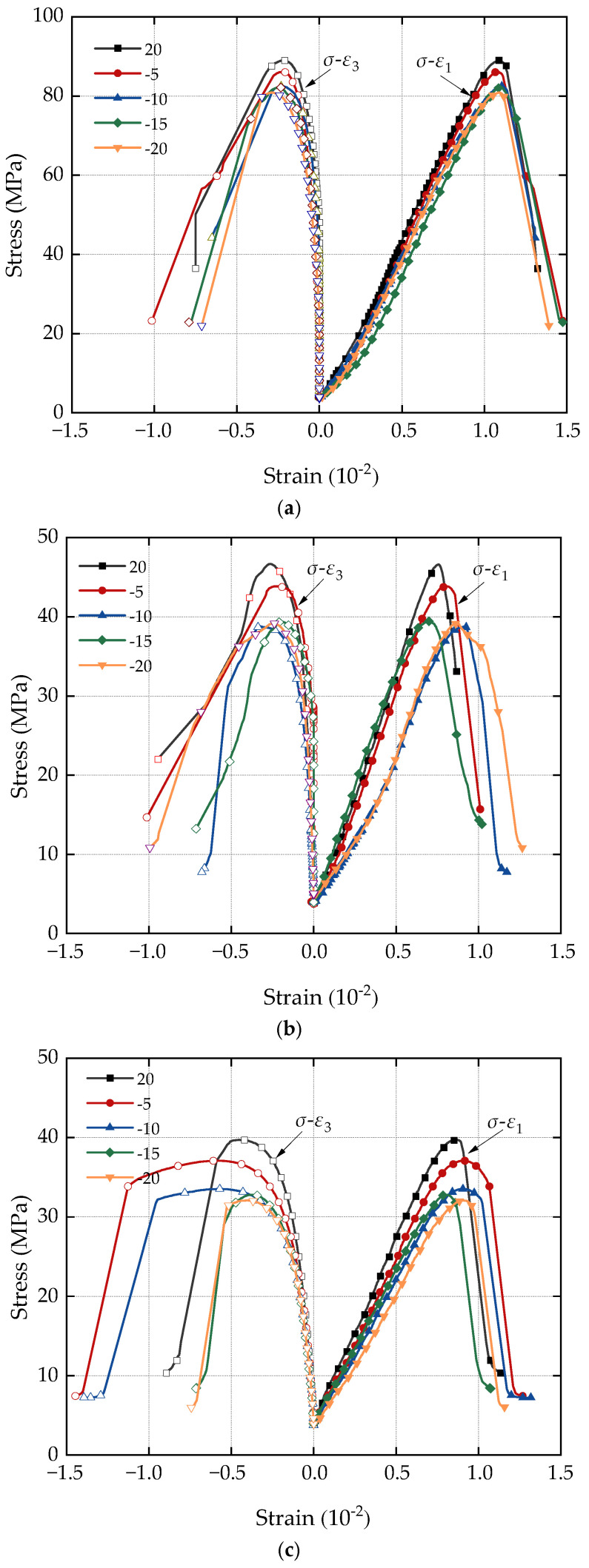
Triaxial compression stress–strain curves of sandstone with different porosity: (**a**) *n* = 3.79%; (**b**) *n* = 11.61%; (**c**) *n* = 16.21%.

Figure 3 depicts the stress–strain curves of three types of porous sandstone undergoing hydraulic coupling at various negative temperatures following a single freeze–thaw test under triaxial compression. Notably, the peak deviatoric stress for all three types of sandstone decreased, with the attenuation amplitude exhibiting a positive correlation with the porosity of the sandstones under different negative temperature freeze–thaw conditions.

For instance, in the sandstone sample group with smaller porosity (*n* = 3.79%), the triaxial compression stress–strain curve after one freeze–thaw cycle follows a similar trend to the stress–strain curve of the sandstone sample at room temperature. The change in peak deviatoric stress is relatively minor in this scenario. However, in the sandstone sample group with porosity *n* = 11.61% and *n* = 16.21%, the peak deviator stress of the sample attenuates significantly in the range of freezing and thawing temperature decreasing from room temperature to −10 °C, while the changes in other situations are not significant.

**Figure 4:**
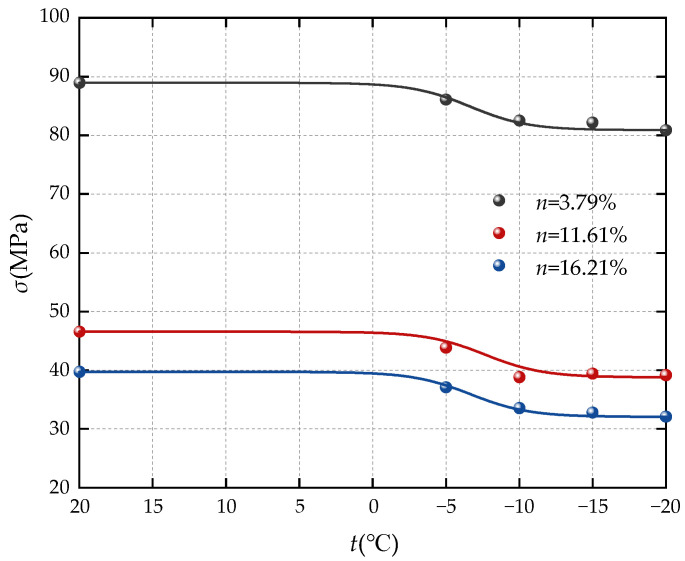
Relationship curve between peak deviatoric stress and freezing temperature of sandstone.

Figure 4 illustrates the relationship between the peak deviatoric stress of sandstone with different porosities and the variation in freezing temperature. The chart reveals the following observations: (1) The peak deviator stress of sandstones with different porosities decreased from 88.94 MPa, 46.56 Mpa, and 39.72 Mpa at room temperature to 80.88 Mpa, 39.16 Mpa, and 32.08 Mpa at −20 °C, indicating a consistent decreasing trend after undergoing a freeze–thaw cycle. (2) The peak deviatoric stress of the sandstones experiences a significant decrease from room temperature to −10 °C, with the attenuation becoming less pronounced within the range of −10 °C to −20 °C. This suggests that the freezing temperature has a considerable impact on the triaxial compressive strength of sandstone after thawing, especially at temperatures of −5 °C and −10 °C. When the freezing temperature drops below −10 °C, the decrease in the peak strength of sandstone slows down significantly. The primary reason for the strength attenuation post-freeze–thaw lies in the frost heave effect during freezing, which weakens the bonding state between sandstone particles. As the freezing process unfolds, free water in the external pores freezes first, isolating the connection between the internal and external water of the sample. As the freezing process progresses, unfrozen water migrates to the interior of the sample, causing the central water pressure of the sample to increase, further expanding the small-pore cracks, and ultimately completely freezing the high-pressure area in the center of the sample and the free water in the smaller pores. Upon melting, the frost heave effect subsides, leading to a decrease in internal pressure within pores and cracks. The effect of geostress and pore water pressure weakens the rock cementation ability, resulting in significant peak deviatoric stress attenuation in sandstone samples, particularly at higher freezing temperatures.

**Figure 5:**
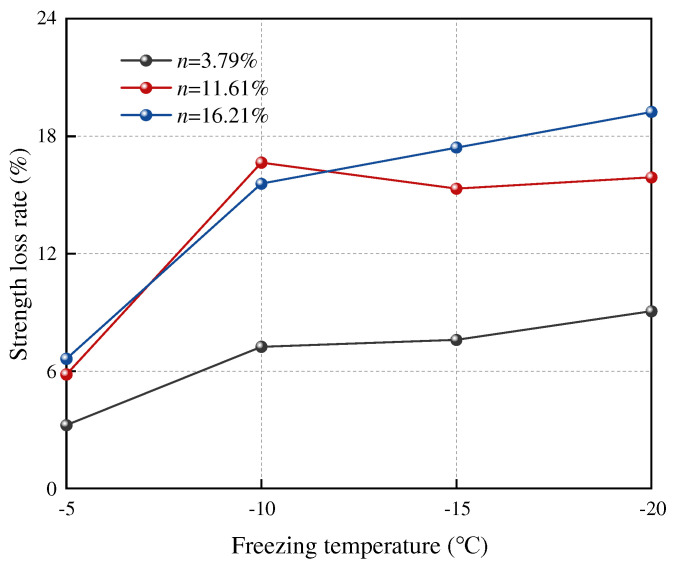
Relationship curve between sandstone strength loss rate and freezing temperature.

From the above analysis, it can be seen that the peak deviatoric stress of sandstone samples decreases to varying degrees before and after freeze–thaw cycles. Therefore, the reduction in compressive strength after a freeze–thaw cycle is used to describe the freeze–thaw damage of saturated sandstone samples. The relationship between the compressive strength loss rate and the freezing temperature is shown in Figure 5.

Figure 5 reveal a noticeable increase in the triaxial compressive strength loss rate of sandstone samples as the freezing temperature decreases. In the experimental group with a porosity of *n* = 3.79%, the triaxial compressive strength loss rate of sandstone rises from 3.23% at −5 °C to 9.06% at −20 °C, reflecting a 5.83% increase. Meanwhile, in the experimental group with a porosity of *n* = 16.21%, the triaxial compressive strength loss rate of sandstone elevates from 6.63% at −5 °C to 19.24% at −20 °C, indicating a 12.61% increase. These results underscore the significant impact of freezing temperature on the triaxial compressive strength of sandstone, with higher initial porosity leading to a greater strength loss rate. When *n* = 3.79%, the strength loss rate at −10 °C reaches 79.9% of the maximum loss rate; when *n* = 11.61%, the strength loss rate at −10 °C reaches 100% of the maximum loss rate; and when *n* = 16.21%, the strength loss rate at −10 °C reaches 80.9% of the maximum loss rate. Combined with Figure 5, it can be found that the strength loss rate of the sandstone sample changes greatly at −5 °C and −10 °C, and the strength loss rate at −15 °C and −20 °C changes less than that at −10 °C, and the change range of the loss rate is also smaller.

### 3.2. Elastic Modulus and Poisson’s Ratio of Sandstone with Different Porosity

**Figure 6:**
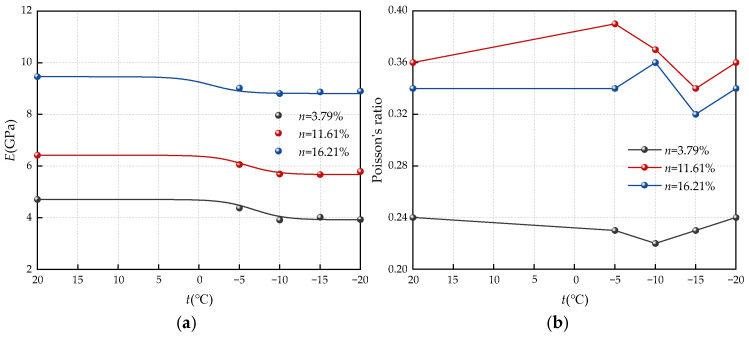
Variation of elastic modulus and Poisson’s ratio with freezing temperature: (**a**) *E*; (**b**) Poisson’s ratio.

Figure 6 illustrates the relationship between the elastic modulus and Poisson’s ratio of the three sandstones and the temperature change during freezing. Key observations from the figure and charts include the following: (1) The elastic modulus of the three types of sandstone (*n* = 3.79%, *n* = 11.61%, and *n* = 16.21%) with different porosities decreases from 4.71 Gpa, 6.42 Gpa, and 9.46 Gpa without freezing to 3.93 Gpa, 5.79 Gpa, and 8.9 Gpa at −20 °C. This indicates a decrease in the elastic modulus of sandstone with varying porosities as the freezing temperature decreases, highlighting the significant impact of freezing temperature on the elastic modulus of sandstone. (2) The Poisson ratio of the three porosity sandstones is minimally affected by freezing temperature, fluctuating within a certain range; notably, the Poisson ratio of high-porosity sandstones is larger than that of low-porosity sandstones. (3) The elastic modulus of the three sandstones decreased significantly from room temperature to −5 °C, decreasing by 2.33%, 6.11%, and 10.5%, respectively, and then further decreased from −10 °C to −20 °C. The results suggest that the temperature during freezing has a pronounced effect on the elastic modulus of sandstone after melting, particularly at −5 °C. Beyond −10 °C, the freezing temperature has a diminished impact on the elastic modulus of sandstone. The attenuation pattern of the elastic modulus in sandstone samples mirrors that of peak deviatoric stress.

### 3.3. Evolution Characteristics of Sandstone Pore Structure Based on Nuclear Magnetic Resonance (NMR) Technology

To examine the alterations in the pore structure of sandstone samples before, during, and after freezing, a low-field nuclear magnetic resonance analysis system was employed. Pore structure scanning tests were conducted on sandstone samples with varying porosities (*n* = 3.79%, 11.61%, and 16.21%) in the pre-freezing, freezing, and post-thawing stages. The changes in the transverse relaxation time (T2) spectrum distribution of sandstone samples with different porosities during various freezing stages were compared and analyzed, shedding light on the damage evolution mechanism of the sandstone pore structure.

#### 3.3.1. Changing Law of Nuclear Magnetic Porosity of Sandstone

The primary principle of nuclear magnetic resonance measurement of rock porosity relies on determining the total number of hydrogen nuclei in the sample. Since nuclear magnetic resonance measures the porosity of the same sample at different stages, nuclear magnetic porosity can directly reflect the evolution process of porosity during the freezing process of sandstone. Table 2 presents the nuclear magnetic porosity of three types of sandstone samples with different porosity before freezing, after freezing, and after thawing, along with the change rate of nuclear magnetic porosity after freezing and thawing compared to that before freezing.

Observations from the table include the following: (1) Among the tested rock samples with different porosities, the nuclear magnetic porosity of the samples increased after freezing and thawing compared to before freezing, but the increase varied. For instance, the nuclear magnetic porosity of N-1 samples increased by 1.25% and 0.63% after freezing and thawing, with change rates of 13.09% and 6.54%, respectively. N-5 rock samples exhibited the lowest change rates, with increases of 4.66% and 1.70% after freezing and thawing. (2) The change rate of nuclear magnetic porosity after freezing and thawing for sandstone samples with small porosity is generally higher than that for sandstone with large porosity, indicating a negative correlation between the change rate of nuclear magnetic porosity after freezing and thawing and porosity. (3) The nuclear magnetic porosity in frozen sandstone is greater than that after thawing, suggesting that the volume of pores in frozen sandstone is larger than that after thawing.

The NMR-measured porosity primarily results from the summation of water signals within cracks of different sizes. Negative-temperature freezing causes the originally present free water in the pores to freeze and solidify, leading to volume expansion. This expansion exerts pressure on the particle surface in the pore wall, enlarging the volume occupied by the pores and even creating new tiny cracks. This process is the fundamental reason for the increase in rock porosity after freezing. Upon thawing, the frozen ice in the pores undergoes liquefaction, resulting in volume reduction and the withdrawal of pressure on the pore wall. The tiny cracks caused by frost heave close again, and the volume of larger pores diminishes with the disappearance of frost heave. Consequently, the nuclear magnetic porosity of the sample after melting is smaller than during the freezing process. Additionally, during vacuum saturation of the melted sandstone, free water re-enters the nascent cracks and tiny pores due to pressure. This action results in an increase in nuclear magnetic porosity during detection.

#### 3.3.2. NMR T2 Spectrum Analysis of Sandstone

**Figure 7:**
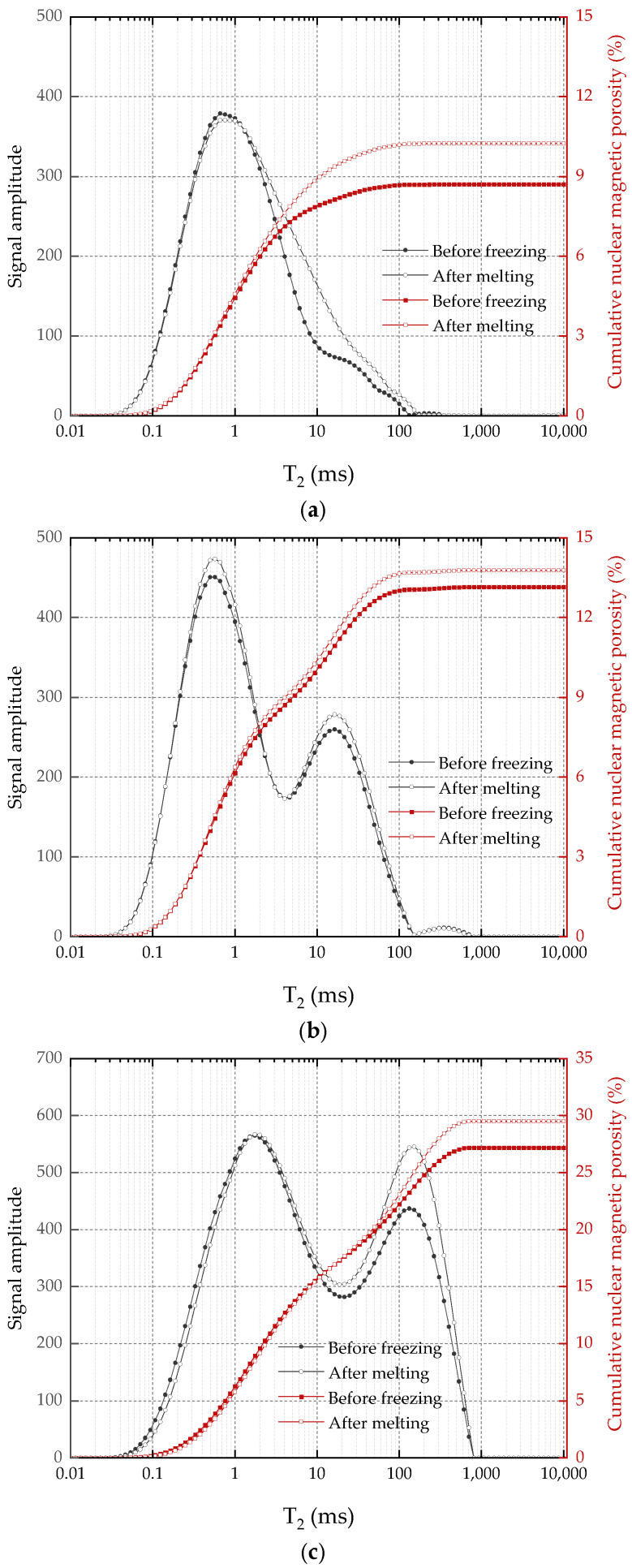
T2 spectrum distribution curve and cumulative nuclear magnetic porosity of sandstone before and after freezing: (**a**) *n* = 3.79%; (**b**) *n* = 11.61%; (**c**) *n* = 16.21%.

The T2 value corresponds to pore size, and the T2 value distribution reflects information about the pore structure inside the rock sample. Changes in the T2 spectrum of sandstone samples after freezing and thawing indicate the impact of freezing on the evolution of the pore structure. Figure 7a–c depict the T2 spectral distribution curves and cumulative nuclear magnetic porosity of rock samples with three porosity levels before and after freezing, respectively. Observations from the figures include the following:

(1) For the small initial porosity (*n* = 3.79%), as shown in Figure 7a, after the sandstone sample undergoes freeze–thaw cycles, the first peak dominates the T2 spectrum, with the second peak contributing minimally. This suggests a scarcity of large and medium-sized pores in sandstone with small porosity, where small and medium-sized pores constitute the main components of the pore structure. Compared to the T2 curve of sandstone before freezing, the post-thaw curve notably changes on the right side of the first spectral peak, generally surpassing the pre-freezing curve. This indicates an increase in the number of corresponding-size pores on the right side of the spectral peak after freeze–thaw cycles. On the left side of the first spectral peak, T2 curves of rock samples before and after freezing largely coincide, indicating minimal impact on rocks with small pores due to freeze–thaw action.
(2) For an initial porosity of *n* = 11.61%, as shown in Figure 7b, the first two spectral peaks of T2 distribution increase after freeze–thaw cycles, and the curves on both sides of the peaks coincide. This suggests an increase in the number of small and medium-sized pores in the rock sample due to freeze–thaw action, causing damage to the pore structure.
(3) When the initial porosity is 16.21%, the second peak value of T2 distribution significantly surpasses the pre-freezing peak value after freeze–thaw cycles. As T2 is proportional to the pore size (r), this indicates a substantial increase in the number of large-size pores in the rock sample with large porosity due to freeze–thaw action. With the increase in T2 value, the signal amplitude value of the rock sample after thawing appears to shift to the right. This phenomenon occurs because smaller-sized pores develop into larger ones under frost heave, causing the value at the front end of the T2 curve to decrease after thawing while the value at the end increases.

### 3.4. Statistical Damage Constitutive Model

#### 3.4.1. Construction of Statistical Damage Constitutive Model

During the formation of rocks over a long period of time, there are various defects in the interior, including various unevenly distributed through-cracks and pores, which provide a place for the flow of groundwater in the underground aquifer. In the complex underground pressure environment, ground stress and pore water pressure have different degrees of complex effects on the failure of porous rocks. In order to explore the damage evolution mechanism of sandstone before and after freeze–thaw cycling under complex stress, based on the existing research results, this section established a statistical damage constitutive model that considers the comprehensive effects of freeze–thaw damage, ground stress, and pore water pressure.

First, the influence of pore water pressure is considered. This paper refers to the statistical damage model of rock considering pore water pressure obtained by Wang et al. [28] based on the effective stress principle and statistical damage theory:

(1) $$\sigma_{1t}=\left[E\varepsilon_{1t}+\left(1-2\mu \right)\left(\sigma_{3}-P_{w}\right)\right]\cdot \exp\left[-{\left(\frac{F}{F_{0}}\right)}^{m}\right]+\left(2\mu -1\right)\left(\sigma_{3}-P_{w}\right)$$

(2) $$F=\frac{\left[E\varepsilon_{1t}+\left(1-2\mu \right)\left(\sigma_{3}-P_{w}\right)\right]}{\sigma_{1t}+\left(1-2\mu \right)\left(\sigma_{3}-P_{w}\right)}\cdot \left[\frac{\sin\varphi \left(\sigma_{1t}+3\sigma_{3}-3P_{w}\right)}{\sqrt{9+3\sin^{2}\varphi }}+\frac{\sigma_{1t}}{\sqrt{3}}\right]$$

where *σ*_1*t*_ is the axial deviator stress, *E* and *μ* are elastic modulus and Poisson’s ratio respectively, *ε*_1*t*_ is the actual strain measured in the experiment, *P_w_* is the pore water pressure, *F* represents the random distribution variable of rock element strength, *F*_0_, *m* are Weibull parameters, and *φ* is the internal friction angle of the rock.

In the coupling triaxial compression test of in situ stress and pore water pressure, the sample is first loaded with simulated in situ stress, and the values of axial pressure and confining pressure are different. Then, pore water pressure is loaded. The processing of rock stress–strain relationship starts after simulating the environmental stress on the sample, but in fact, the initial axial strain of the sample has already occurred during the loading process of in situ stress. According to the effective stress principle and generalized Hooke’s law, the following can be concluded:

(3) $$\varepsilon_{10}=\frac{1}{E}\left[\sigma_{10}-2\mu \sigma_{3}-\left(1-2\mu \right)P_{w}\right]$$

where *σ*_l0_ is the simulated vertical geostress on the rock, and the actual strain *ε*_l_ that occurs in the axial direction of the rock is the sum of the experimental measurement values *ε*_l*t*_ and the axial strain *ε*_l0_ that occurs during the simulated geostress process, namely:

(4) $$\varepsilon_{1}=\varepsilon_{10}+\varepsilon_{1t}$$

By bringing Equations (3) and (4) into Equations (1) and (2), the statistical damage constitutive model of sandstone considering pore water pressure and in situ stress conditions can be obtained:

(5) $$\sigma_{1t}=\left[E\varepsilon_{1t}+\sigma_{10}-2\mu \sigma_{3}-\left(1-2\mu \right)P_{w}\right]\cdot \exp\left[-{\left(\frac{F}{F_{0}}\right)}^{m}\right]+\left(2\mu -1\right)\left(\sigma_{3}-P_{w}\right)$$

(6) $$F=\frac{E\varepsilon_{1t}+\sigma_{10}-2\mu \sigma_{3}-\left(1-2\mu \right)P_{w}}{\sigma_{1t}+\left(1-2\mu \right)\left(\sigma_{3}-P_{w}\right)}\cdot \left[\frac{\sin\varphi \left(\sigma_{1t}+3\sigma_{3}-3P_{w}\right)}{\sqrt{9+3\sin^{2}\varphi }}+\frac{\sigma_{1t}}{\sqrt{3}}\right]$$

Assuming that rock mass failure follows a Weibull distribution, the damage variable *D* of rock under load can be expressed as follows:

(7) $$D=1-\exp\left[-{\left(\frac{F}{F_{0}}\right)}^{m}\right]$$

Due to freeze–thaw action, damage has occurred inside the rock, so the two damage factors of freeze–thaw cycling and loading should be considered comprehensively to establish the damage constitutive model.

The freeze–thaw damage variable *D_r_* is defined by the elastic modulus of rock before and after freeze–thaw cycling, and can be expressed as follows:

(8) $$D_{r}=1-\frac{E_{r}}{E_{0}}$$

where *E_r_* is the elastic modulus of the rock after one freeze–thaw cycle, and *E*_0_ is the elastic modulus of the rock that has not undergone freeze–thaw cycles.

Considering the above factors, the total damage variable *D_t_* of rock subjected to freeze–thaw cycling and loading damage can be expressed as follows:

(9) $$D_{t}=1-\left(1-D_{r}\right)\left(1-D\right)$$

(10) $$D_{t}=D_{r}+D-D_{r}D$$

Combining Equations (7) and (8), the damage variable of sandstone after freeze–thaw cycling can be expressed as follows:

(11) $$D_{t}=1-\frac{E_{r}}{E_{0}}\exp\left[-{\left(\frac{F}{F_{0}}\right)}^{m}\right]$$

According to Equation (5), the statistical damage constitutive model of sandstone after a freeze–thaw process can be obtained considering pore water pressure and geostress conditions:

(12) $$\sigma_{1t}=\left[E\varepsilon_{1t}+\sigma_{10}-2\mu \sigma_{3}-\left(1-2\mu \right)P_{w}\right]\frac{E_{r}}{E_{0}}\cdot \exp\left[-{\left(\frac{F}{F_{0}}\right)}^{m}\right]+\left(2\mu -1\right)\left(\sigma_{3}-P_{w}\right)$$

In the early stage of stress loading on rocks, various internal defects such as cracks and pores will compress and close, appearing as concave shapes in the front of the stress–strain curve. Considering the compaction process, the compaction coefficient *K* [29] is introduced for correction:

(13) $$K=\left\{\begin{array}{ll} \log_{n}\left[\frac{\left(n-1\right)\varepsilon }{\varepsilon_{y}}+1\right] & \left(\varepsilon \leq \varepsilon_{y}\right)\\ 1 & \left(\varepsilon \geq \varepsilon_{y}\right) \end{array}\right.$$

where *ε_y_* is the axial strain corresponding to the peak stress of the sandstone sample, and n is the constant parameter obtained from the experimental data. After adding the compaction factor, Equation (13) can be expressed as follows:

(14) $$\sigma_{1t}=\left[KE\varepsilon_{1t}+\sigma_{10}-2\mu \sigma_{3}-\left(1-2\mu \right)P_{w}\right]\frac{E_{r}}{E_{0}}\cdot \exp\left[-{\left(\frac{F}{F_{0}}\right)}^{m}\right]+\left(2\mu -1\right)\left(\sigma_{3}-P_{w}\right)$$

#### 3.4.2. Determination of Model Parameters

In the established statistical damage constitutive model for sandstone after a single freeze–thaw cycle, the parameters to be determined are *m* and *F*_0_, while the remaining parameters can be obtained through experimental data processing. Equation (14) can be transformed into the following:

(15) $$\left[\frac{\sigma_{1t}-\left(2\mu -1\right)\left(\sigma_{3}-P_{w}\right)}{E\varepsilon_{1t}+\sigma_{10}-2\mu \sigma_{3}-\left(1-2\mu \right)P_{w}}\right]\frac{E_{0}}{E_{r}}=\exp\left[-{\left(\frac{F}{F_{0}}\right)}^{m}\right]$$

Taking two logarithms on both sides of the Equation (16) above gives:

(16) $$\ln\left\{\ln\left[\frac{E\varepsilon_{1t}+\sigma_{10}-2\mu \sigma_{3}-\left(1-2\mu \right)P_{w}}{\sigma_{1t}-\left(2\mu -1\right)\left(\sigma_{3}-P_{w}\right)}\frac{E_{r}}{E_{0}}\right]\right\}=\mathrm{mln}F-\mathrm{mln}F_{0}$$

Let

(17) $$Y=\ln\left\{\ln\left[\frac{E\varepsilon_{1t}+\sigma_{10}-2\mu \sigma_{3}-\left(1-2\mu \right)P_{w}}{\sigma_{1t}-\left(2\mu -1\right)\left(\sigma_{3}-P_{w}\right)}\frac{E_{r}}{E_{0}}\right]\right\}$$

(18) X=lnF

(19) $$B=m\ln F_{0}$$

Equation (16) can be transformed into the following:

(20) Y=m·X−B

Based on experimental data, obtain *Y* and *X* values and perform linear fitting to obtain *m* and *B* values. Then, according to the equation

(21) $$F_{0}=\exp\left(B/m\right)$$

the value of parameter *F*_0_ can be obtained, which can then determine the statistical damage constitutive model of rocks under the action of in situ stress and pore water pressure after a single freeze–thaw cycle. The parameters of the statistical damage constitutive model for sandstone with different porosity values after four kinds of cryogenic freezing and thawing are shown in Table 3.

#### 3.4.3. Verification of the Statistical Damage Composition Model

**Figure 8:**
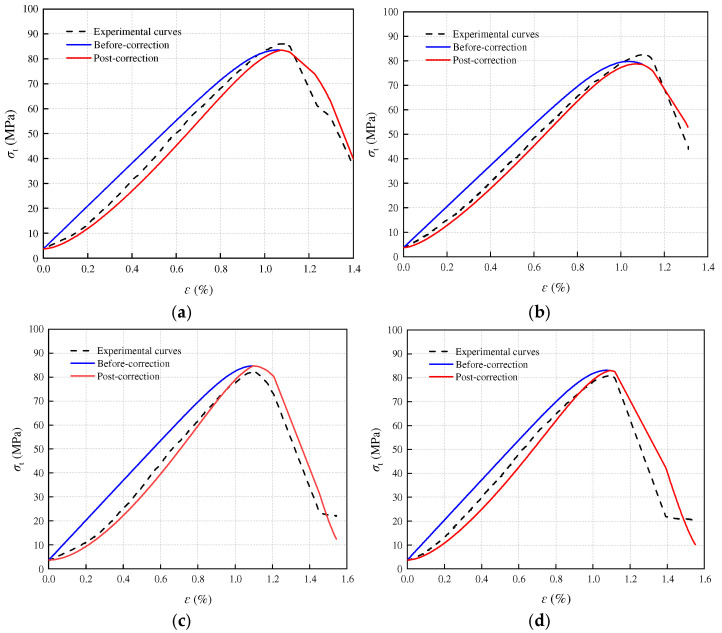
Porosity 3.79% sandstone statistical damage constitutive model validation: (**a**) T = −5 °C; (**b**) T = −10 °C; (**c**) T = −15 °C; (**d**) T = −20 °C.

**Figure 9:**
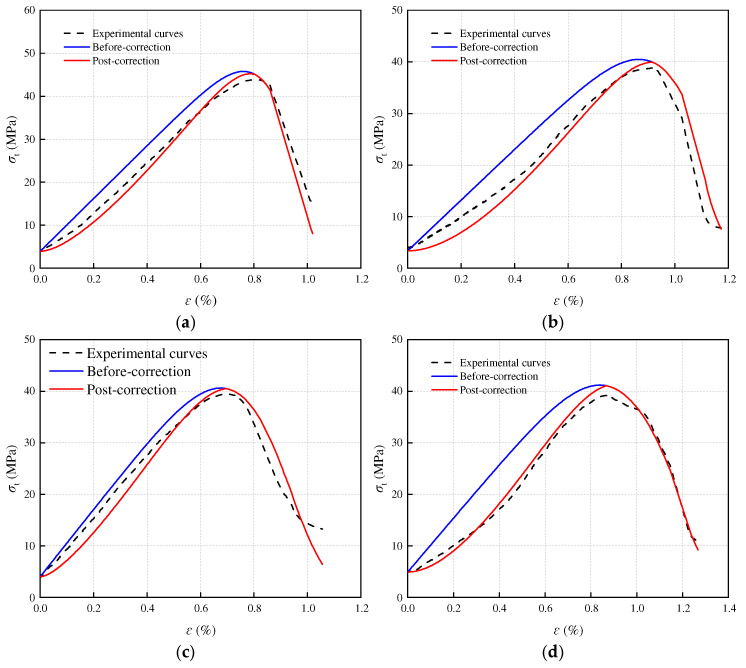
Porosity 11.61% sandstone statistical damage constitutive model validation: (**a**) T = −5 °C; (**b**) T = −10 °C; (**c**) T = −15 °C; (**d**) T = −20 °C.

**Figure 10:**
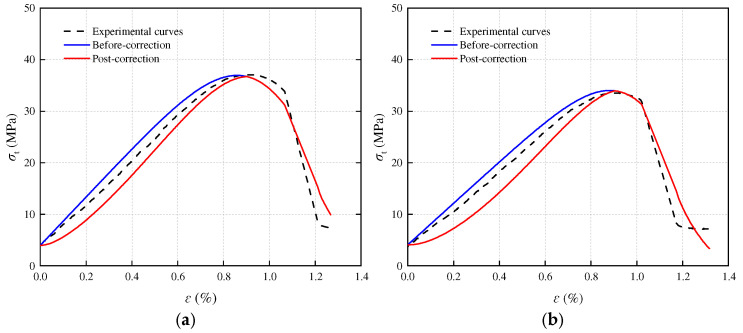

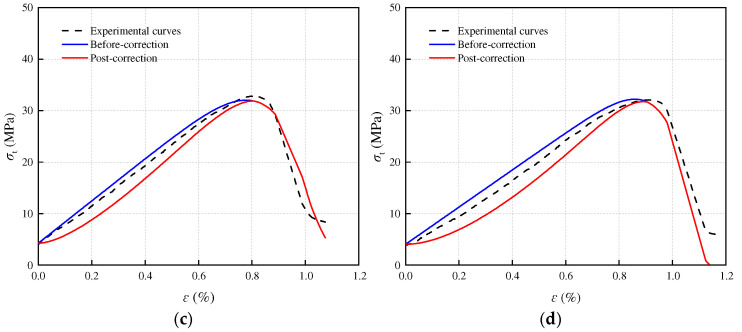
Porosity 16.21% sandstone statistical damage constitutive model validation: (**a**) T = −5 °C; (**b**) T = −10 °C; (**c**) T = −15 °C; (**d**) T = −20 °C.

Figure 8, Figure 9 and Figure 10 present a comparison between the theoretical and experimental curves of three constitutive models applied to sandstone with varying porosities. Observing the figures, it becomes evident that the experimental curves for porosities of 3.79% and 11.67% align more closely with the theoretical model curve that includes an additional compaction coefficient *K*. Conversely, the experimental curve for sandstone with a porosity of 16.21% more closely resembles the theoretical model curve without the added compaction coefficient *K*. This observation may stem from the fact that high-porosity sandstone samples exhibit weak inter-particle cementation and lower rock strength. Following freeze–thaw damage, internal damage intensifies, leading to a higher compression degree in the early stage of loading failure for high-porosity sandstone samples due to simulated in situ stress and pore water pressure loading. Consequently, the stress–strain curve obtained from the experiment directly transitions through the compaction stage into the elastic deformation stage. However, the simulated in situ stress loading did not fully compact the other two samples with smaller porosities, and these samples underwent a compaction process during the loading failure stage, bringing them closer to the theoretical model with a compaction coefficient *K*.

In summary, the statistical damage constitutive model, incorporating the freeze–thaw damage variable *D_r_*, proves to be more effective in characterizing the stress–strain relationship of sandstone under the complex conditions of freeze–thaw action, in situ stress, and pore water pressure.

### 3.5. Discussion

This study focuses on the effects of freeze–thaw cycles on the mechanical properties and pore structure evolution of sandstone, and proposes some new insights by combining experimental results and statistical damage constitutive models. Although the results have high scientific and application value, further analysis and discussion will help deepen the research significance and reveal future research directions.

Through freeze–thaw experiments, it was found that the peak deviatoric stress and elastic modulus both decreased with the decrease in freezing temperature, especially in the range of −5 °C to −10 °C, and tended to be stable below −10 °C. This phenomenon may be closely related to the frost heave effect. When the temperature is above −10 °C, the frost heave of water in the pores has a strong destructive effect on the binding force between rock particles; when it is below −10 °C, further temperature reduction mainly causes the already frozen pore water to solidify, and the generation of new cracks is limited. Nuclear magnetic resonance analysis shows that the porosity changes in sandstone with lower porosity after freezing and thawing are more significant. The reason for this may be that the small-pore samples are more significantly affected by the frost heave pressure, and the internal cracks expand more efficiently, resulting in a rapid increase in porosity. However, the samples with higher porosity have a relatively small change range due to the larger original pore space and limited pore expansion. This discovery provides a new perspective for understanding the evolution mechanism of sandstone damage under different initial porosity conditions. The damage constitutive model proposed in this study combines the coupling effects of freeze–thaw cycles, geostress, and pore water pressure well, and systematically describes the complexity of sandstone mechanical behavior. The good agreement between the experimental results and the theoretical model curve proves the effectiveness of the model, especially in small-porosity and medium-porosity samples. However, for high-porosity sandstone, the fitting accuracy between the theoretical curve and the experimental curve is low in the initial compaction stage, which may be related to the failure to completely compact the high-porosity sample when simulating geostress loading. In the future, the model can be further optimized by introducing correction parameters such as pore distribution uniformity or freeze–thaw cycle number.

The research results have important guiding significance for engineering practices in complex frozen soil environments such as shaft wall design and tunnel construction. However, the samples used in the study only include sandstones with three porosities, and the experimental conditions are a single freeze–thaw cycle, which limits the wide applicability of the model. For example, the cumulative effect of freeze–thaw cycles and the influence of acidic or saline water environments on the pores and mechanical properties of sandstone have not been involved. In addition, although low-field nuclear magnetic resonance technology is more sensitive to the detection of pore structure changes, its combined analysis with other microscopic detection techniques (such as CT scanning) can help further verify the reliability of the experimental results. This study provides a new theoretical basis for the evolution of mechanical damage of sandstone under freeze–thaw conditions, but further exploration is needed in the future. On the one hand, the experimental conditions can be extended to multiple freeze–thaw cycles, different water chemical environments, or more complex temperature change conditions; on the other hand, the freeze–thaw damage behavior of other rock types can be considered to improve the universality of the model. At the same time, combining macroscopic mechanical experiments with microscopic detection methods, such as scanning electron microscopy (SEM) or X-ray diffraction (XRD), can further reveal the intrinsic mechanism of freeze–thaw damage.

In summary, the experimental results and model verification of this study provide important data support for the study of the mechanical behavior of sandstone in freeze–thaw environments. Through the deepening of subsequent research and optimization of models, this field will better serve actual engineering needs.

## 4. Conclusions

To investigate the mechanical properties of sandstone under the coupling of water pressure and confining pressure after a single freeze–thaw cycle, water-force coupling triaxial compression tests were conducted on sandstone having three types of porosity at various negative temperatures. The effects of porosity and freezing temperature on the compressive strength and elastic modulus of sandstone were studied. Microscopic damage characteristics and the damage evolution mechanism of sandstone were analyzed through nuclear magnetic resonance testing and a statistical damage constitutive model. The main conclusions are outlined below:

(1) The stress–strain curve of sandstone samples under triaxial compression after freeze–thaw shows little change compared to that without freezing at normal temperatures. The peak deviator stress exhibits a decreasing trend with the reduction in freezing temperature, particularly pronounced between −5 °C and −10 °C, with minor influence when the temperature drops below −10 °C.
(2) The overall elastic modulus of sandstone with different porosities decreases with decreasing freezing temperature. The elastic modulus experiences a significant decrease from −5 °C to −10 °C. Below −10 °C, the elastic modulus remains similar to that at −10 °C, showing little change. The relationship between Poisson’s ratio and the freezing temperature for three porosities of sandstone is irregular.
(3) Nuclear magnetic porosity of sandstone samples significantly increases after both freezing and thawing processes. The smaller the initial porosity of the rock sample, the greater the rate of change in nuclear magnetic porosity after freeze–thaw cycling. Differences exist in the influence of freeze–thaw damage on the T2 distribution of sandstone with different porosities.
(4) A statistical damage constitutive model, considering the combined effects of freeze–thaw damage, geostress, and pore water pressure, was established. The compaction coefficient *K* was introduced into the model for optimization. The theoretical curve and experimental curve trends are relatively close, providing a better characterization of the stress–strain relationship of sandstone in complex pressure environments.
(5) Although this study systematically analyzed the mechanical properties and damage evolution mechanism of sandstone under freeze–thaw action and established a statistical damage constitutive model, there are still some limitations. First, the experiment only targeted sandstone samples with three porosities, and the number of freeze–thaw cycles was limited, which failed to fully reflect the cumulative effect of multiple freeze–thaw cycles on the mechanical properties of sandstone. In addition, the research environment was mainly based on a single hydraulic coupling condition, and the potential influence of water chemistry (such as acidity or salinity) on sandstone damage was not considered. Although low-field nuclear magnetic resonance technology reveals the evolution law of the pore structure, the joint verification with other microscopic detection methods is still insufficient, which may limit the comprehensiveness of the results. Future research can be expanded in terms of the following aspects: first, increase the number of freeze–thaw cycles and consider the long-term effects of different environmental conditions (such as acidic water or salt freezing) on sandstone damage; second, conduct comparative studies on multiple rock types to improve the applicability and universality of the model; and third, combine more accurate microscopic detection techniques (such as scanning electron microscopy and CT scanning) to deeply reveal the freeze–thaw damage mechanism from a multi-scale level. Through the above improvements, the research results will better serve the engineering design and maintenance in complex geological environments.

## Figures and Tables

**Table 1:** Basic physical parameters of sandstone samples.

Sample	Dry Density (g/cm^3^)	Saturation Density (g/cm^3^)	Porosity *n* (%)	UCS (MPa)
A	2.37	2.41	3.79	76.59
B	2.23	2.33	11.61	29.69
C	2.16	2.32	16.21	18.99

**Table 2:** Nuclear magnetic porosity and change rate of sandstone under different conditions.

Porosity (%)	Rock Specimen	Nuclear Magnetic Porosity (%)			Nuclear Magnetic Porosity Change Rate (%)	
		Before Freezing	In Freezing	After Melting	In Freezing	After Melting
3.79	N-1	9.58	10.83	10.21	13.09	6.54
	N-2	8.50	9.28	9.01	9.25	6.10
11.61	N-3	13.15	14.28	13.78	8.64	4.77
	N-4	19.57	20.48	20.21	4.63	3.26
16.21	N-5	29.00	30.36	29.50	4.66	1.70
	N-6	30.89	32.44	31.70	5.01	2.61

**Table 3:** Parameters of statistical damage constitutive model of sandstone under in situ stress and pore water pressure.

Porosity (%)	Freezing Temperature (°C)	Elastic Modulus (GPa)	Poisson’s Ratio	Peak Strain (%)	*F*_0_	*m*
3.79	−5	9.021	0.233	1.0743	99.564	9.049
	−10	8.813	0.220	1.1061	97.211	9.175
	−15	8.867	0.225	1.097	96.438	9.832
	−20	8.903	0.215	1.0925	95.645	9.257
11.61	−5	6.059	0.392	0.8041	54.655	6.646
	−10	5.693	0.373	0.9164	53.306	7.452
	−15	5.667	0.343	0.6964	50.886	6.672
	−20	5.790	0.352	0.8664	50.642	5.283
16.21	−5	4.375	0.340	0.9028	47.580	6.629
	−10	3.919	0.363	0.9043	45.323	7.347
	−15	4.016	0.316	0.8057	41.711	7.360
	−20	3.926	0.301	0.9043	41.358	7.931

## Data Availability

The original contributions presented in the study are included in the article, further inquiries can be directed to the corresponding author.
